# DNA Damage-Inducing Anticancer Therapies: From Global to Precision Damage

**DOI:** 10.3390/cancers12082098

**Published:** 2020-07-28

**Authors:** Thom G. A. Reuvers, Roland Kanaar, Julie Nonnekens

**Affiliations:** 1 Department of Molecular Genetics, Erasmus MC, Dr. Molenwaterplein 40, 3015 GD Rotterdam, The Netherlands; t.reuvers@erasmusmc.nl (T.G.A.R.); r.kanaar@erasmusmc.nl (R.K.); 2 Department of Radiology and Nuclear Medicine, Erasmus MC, Dr. Molenwaterplein 40, 3015 GD Rotterdam, The Netherlands; 3 Oncode Institute, Office Jaarbeurs Innovation Mile (JIM), Jaarbeursplein 6, 3561 AL Utrecht, The Netherlands

**Keywords:** DNA damage-inducing therapies, cancer therapy, DNA damage response, DNA repair, radiotherapy, cytotoxic chemotherapy, DDR modulators, combination therapies

## Abstract

DNA damage-inducing therapies are of tremendous value for cancer treatment and function by the direct or indirect formation of DNA lesions and subsequent inhibition of cellular proliferation. Of central importance in the cellular response to therapy-induced DNA damage is the DNA damage response (DDR), a protein network guiding both DNA damage repair and the induction of cancer-eradicating mechanisms such as apoptosis. A detailed understanding of DNA damage induction and the DDR has greatly improved our knowledge of the classical DNA damage-inducing therapies, radiotherapy and cytotoxic chemotherapy, and has paved the way for rational improvement of these treatments. Moreover, compounds targeting specific DDR proteins, selectively impairing DNA damage repair in cancer cells, form a promising novel therapy class that is now entering the clinic. In this review, we give an overview of the current state and ongoing developments, and discuss potential avenues for improvement for DNA damage-inducing therapies, with a central focus on the role of the DDR in therapy response, toxicity and resistance. Furthermore, we describe the relevance of using combination regimens containing DNA damage-inducing therapies and how they can be utilized to potentiate other anticancer strategies such as immunotherapy.

## 1. Introduction

In the clinical landscape of anticancer strategies, DNA damage-inducing therapies have held a prominent position for decades. This is emphasized by the fact that two of the main pillars of cancer treatment, chemotherapy and radiotherapy, exert their anticancer activity by directly or indirectly inducing DNA damage. Apart from these classical therapies, more targeted approaches such as inhibitors of DNA damage-counteracting enzymes are now also entering the clinic [1]. The underlying success of DNA damage-inducing therapies is the rationale that cancer cells respond to DNA damage in a coordinated manner and, under suitable pharmacological conditions, can elicit a variety of responses such as inhibition of cancer cell proliferation and induction of cell death. Alternatively, the induced DNA damage can be repaired in a tightly controlled fashion, counteracting the effects of DNA damage-inducing therapies and possibly even leading to therapy resistance. The induction of DNA damage repair and cellular fates such as proliferation inhibition are orchestrated by a heavily regulated network of proteins, collectively called the DNA damage response (DDR) [2]. Both healthy and cancer cells employ the DDR as a protection mechanism as their DNA is constantly challenged by endogenous- [e.g., reactive oxygen species (ROS)] and exogenous [e.g., ultraviolet (UV) radiation] sources [3]. With regard to therapy-induced DNA damage in cancer cells, DDR pathways thus play a central role in tumor cell responses to DNA damage-inducing therapies as both protective and target mechanisms. Although these therapies induce DNA damage in both healthy and cancer cells, cancer cells are preferentially targeted due to specific alterations in their DDR pathways, leading to genome instability [4]. This can have far-reaching effects on cellular behavior, such as an increase in proliferation rate and impaired DNA repair as compared to healthy cells. Consequently, DNA damage, resulting in detrimental cellular fates such as apoptosis, preferentially affects cancer cells.

The field of DNA damage-inducing therapies has seen a tremendous improvement in impact over the last decades [5]. Since the discovery of the anticancer properties of radiotherapy and chemotherapy, a large amount of knowledge was gained on their mechanism of action and clinical effects in different patient groups [6,7]. Detailed insights into the specific components of the DDR that are activated by therapy have resulted in the development of novel treatment opportunities. Furthermore, a better understanding of toxicity and resistance on a molecular, cellular and tissue-level has led to significant therapy refinement [8,9]. Ultimately, the goal of this research is to improve the therapeutic window, defined as the range of drug dosing between the minimal dose for anti-cancer activity and maximum dose for acceptable toxicity. Prompted by these developments, this review gives a broad overview of the currently applied DNA damage-inducing therapies applied in the clinic and their mechanisms of action, with a central focus on the importance of the DDR in therapy response and resistance. Moreover, it will describe how this knowledge is used to further improve anticancer treatment among other rapidly evolving fields such as immunotherapy.

## 2. The DNA Damage Response

As stated above, the role of the DDR for DNA damage-inducing therapies is crucial, as it can counteract therapy effects, but can also be targeted by specific drugs. Therefore, a detailed understanding of the various types of DNA lesions and the dynamics of the specific molecular pathways that they activate is necessary to understand and improve therapy. The following sections will give an overview of the major types of DNA lesions, as well as the corresponding DNA repair pathways.

### 2.1. Types of DNA Damage

**Figure 1:**
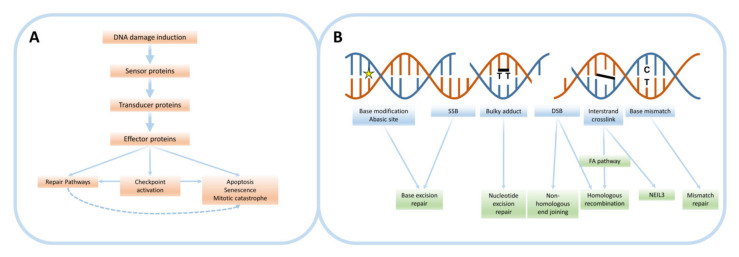
**Overview of the DNA damage response.** (**A**) General overview of the DDR signaling cascade upon DNA damage induction, either endogenously or by external agents. After damage detection by sensor proteins, transducer proteins activate effector proteins that can elicit a variety of cellular responses. Cell cycle checkpoints will be activated to halt proliferation and allow time for (accurate) repair. However, the inability to repair the induced damage can also lead to induction of detrimental cellular fates such as apoptosis. (**B**) Visualization of types of DNA damage that can be induced, as well as the main pathways that are directly involved in their repair. Abbreviations: SSB: single-strand break; DSB: double-strand break; FA pathway: Fanconi anemia pathway.

Various types of DNA lesions can be induced endogenously, either directly or as intermediate structures of repair processes for other lesions, or by external factors such as ionizing radiation (IR) or chemicals (Figure 1B) [10]. First of all, single-strand breaks (SSBs) and double-strand breaks (DSBs) arise from discontinuities in a single DNA strand or both strands, respectively. DSBs are a detrimental form of DNA damage, as even a single DSB is capable of inducing cell death, and their induction and processing can be highly mutagenic and have far-reaching consequences for the genome [11,12]. Importantly, DSBs can also form during cell division, when a replication fork encounters a SSB or a blocking DNA lesion [13]. Furthermore, a wide variety of base and deoxyribose modifications can be introduced, such as abasic sites, formed by the hydrolytic cleavage of a base from a deoxyribose moiety. Additionally, several changes can be made to the base moiety itself, such as deamination, alkylation and the formation of thymine dimers by UV rays [10,14,15]. Moreover, oxidative base or deoxyribose damage can be induced, mainly by reactive oxygen species (ROS), which can either be formed endogenously or as a byproduct from external agents. Other DNA damaging factors can introduce covalent bonds between adjacent nucleotides on either the same or opposing DNA strand(s), termed intrastrand and interstrand crosslinks (ICLs), respectively [16]. Finally, erroneous base incorporation can occur endogenously during DNA replication or can be induced by external agents, leading to base mismatches that distort the DNA helix structure [10].

### 2.2. DNA Damage Repair Pathways

An overview of the general signaling of the DDR is visualized in Figure 1A. Components of the DDR regulate detection, signal transduction and repair upon damage induction and mediate cell cycle progression. After sensor proteins detect DNA damage, cell cycle checkpoints can be activated to halt progression of proliferation and allow time for repair [17]. Recruitment of signal transducer proteins mediates the subsequent activation of cellular repair cascades [2]. However, if DNA repair is unsuccessful or the amount of damage exceeds a certain threshold, this can result in various detrimental cellular fates [18]. These include programmed cell death (apoptosis), stable cellular growth arrest (senescence) and cell death after inappropriate cell division (mitotic catastrophe).

Each type of DNA lesion requires a specific form of repair, performed by different cross-communicating protein cascades. The various repair pathways, along with the specific DNA damage types that activate them, are visualized in Figure 1B. Base excision repair (BER) is the dominating pathway to repair SSBs and base lesions that do not significantly distort the DNA helix structure (reviewed in [19]). General steps of this pathway include flipping out and cleavage of the damaged base, incision of the nucleotide backbone at the resulting abasic site, DNA end processing, and refilling the resulting gap by polymerases followed by ligation. In contrast, lesions that do significantly distort the helix, including thymine dimers and intrastrand crosslinks, are repaired through the nucleotide excision repair (NER) pathway (reviewed in [20]). After detection of these lesions, a single-strand DNA segment around the lesion is removed, after which the remaining single-strand DNA is used as a template for DNA synthesis by polymerases. ICL repair can be mediated by the Fanconi anemia (FA) pathway upon replication fork stalling and subsequent involvement of other repair proteins such as structure-selective endonucleases [21] and those involved in DSB repair (reviewed in [16]). In addition, recently an FA-independent pathway for ICL repair was identified, mediated by the DNA glycosylase NEIL3 [22]. For DSBs a variety of repair cascades have been described (reviewed in [23]). The major generally distinct pathways are homologous recombination (HR) and non-homologous end joining (NHEJ). After damage recognition and end processing, the error-free HR pathway uses homologous DNA on the intact sister chromatid as a template to resynthesize DNA such that the integrity of the broken chromatid can be restored. The requirement of an accessible sister chromatid makes that HR is only effective during the S and G2-phases of the cell cycle. Alternatively, NHEJ is available during all cell cycle stages and involves direct ligation of broken DNA ends without a homologous DNA template. This pathway might therefore result in repair-associated errors such as insertions or deletions. Finally, when erroneous nucleotide incorporation during replication occurs, mismatch repair (MMR, reviewed in [24]) detects a DNA strand containing a mismatched nucleotide. Consequently, the DNA is nicked, excised and resynthesized and ligated using the complementary strand as a template.

## 3. DNA Damage-Inducing Therapies

### 3.1. Radiotherapy

Radiotherapy has been one of the cornerstones of cancer treatment for decades with currently over 50% of cancer patients worldwide receiving this therapy [25]. The most widely used form is external beam radiotherapy (EBRT), which uses high-energy photons with a relatively high tissue penetration depth [26]. EBRT is mainly carried out with a linear accelerator producing x-rays or a cobalt-60 source producing γ-rays [27]. However, other forms of EBRT, especially those employing beams of charged particles such as protons, are becoming increasingly important for cancer treatment [28]. In contrast to EBRT, brachytherapy is practiced by placing the radiation source within the body, close to the tumor [29]. Finally, systemic administration of a radionuclide is used during molecular radionuclide therapy (MRT) [30]. Most research on cellular and physical effects of ionizing radiation (IR) have been performed in the context of EBRT with photon radiation. As the nature of the induced DNA damage is dependent on specific radiation characteristics, the results of this research can probably not be extrapolated one on one to other types of radiation such as proton therapy and MRT. When comparing these different types of radiation, the term relative biological effectiveness (RBE) is often used [31]. This is the ratio of the absorbed dose of a radiation type to the absorbed dose of a reference type that induces the same biological endpoint, with x-rays with a defined energy or cobalt-60 γ-rays often used as the reference radiation type. To exploit the full potential of all these forms of radiotherapy in the near future, more radiobiological research in this area should result in the more accurate prediction of their RBE values and elucidate how their anticancer effect differs from photon beam therapy mechanistically.

On a molecular level, the general effects of IR can be divided in direct and indirect effects (Figure 4). Direct effects result from the physical induction of breaks and other DNA lesions, while indirect effects include ROS formation from intracellular water molecules and subsequent free radical-driven formation of lesions such as abasic sites and SSBs [32]. The BER, HR and NHEJ pathways of the DDR are well-known to play an important role in the cellular response to IR-induced DNA damage. Besides induction of the different single types of DNA damage, groups of multiple lesions in close vicinity (within 1–2 DNA helix turns) have been detected in cells after IR treatment, collectively termed clustered DNA damage [33,34]. These clusters consist of various lesion types, including DSBs, SSBs and abasic sites. The formation of damage clusters is thought to be dependent on the linear energy transfer (LET, defined as the amount of energy a particle disposes along its track per unit of distance) of the IR type used, with higher LETs corresponding to induction of more complex DNA clusters [35]. Importantly, the complexity of DNA damage is suggested to have an effect on DNA repair efficiency. As an example, DSB break termini at clustered DNA damage sites were shown to impair the removal of base lesions by the BER machinery [36]. Findings like these imply that complex DNA damage might contribute significantly to exceeding the cellular capabilities of DNA repair and forces cells towards induction of cell fates such as apoptosis.

Of central importance for the cellular response to IR is the radiosensitivity of targeted cancer cells. This crucial characteristic is influenced by multiple factors, both on a tissue and cellular level. A well-known example of such a factor is tumor hypoxia, which increases radioresistance [37]. Another factor is the cell cycle phase of a cancer cell at the time of DNA damage induction. Cells in the G2/M-phase of the cell cycle are the most radiosensitive, while cells in late S-phase tend to be the most radioresistant [38]. Factors on a DDR-level can also have a significant effect on intrinsic cancer cell radiosensitivity. The way different tumors, or even different cells within the same tumor, deal with IR-induced DNA damage might significantly vary, depending on specific alterations in their DDR. Demonstrating this concept, transfection of tumor cells from DSB repair-deficient mice with DSB repair gene DNA-dependent protein kinase, catalytic subunit (*DNA-PKcs*) increases tumor radioresistance for EBRT [39]. In a more extreme case, mutations in the DSB repair gene ataxia telangiectasia mutated (*ATM*) in the neurodegenerative disease ataxia telangiectasia (AT) leads to hypersensitivity to IR [40]. These findings implicate that DDR alterations can be used as predictive biomarkers for the tumor response to IR [41].

#### 3.1.1. External Beam Radiotherapy

**Figure 2:**
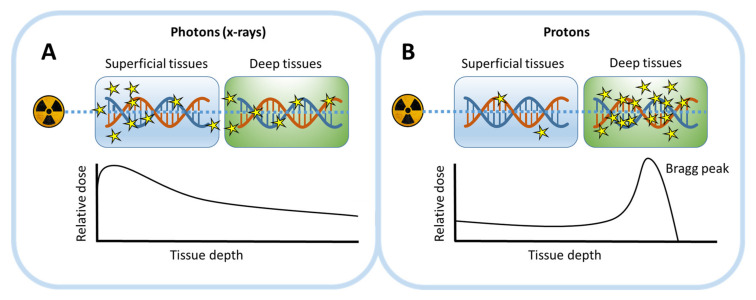
Overview of DNA damage induction patterns as a function of tissue depth for two types of EBRT, using either photon or proton beams. The induction of damage in superficial and deeper tissues is visualized (upper panel), as well as the course of the relative dose with increasing tissue depth (lower panel). (**A**) For photon beams, the highest dose will be deposited at the body entrance site, corresponding with the highest density of induced DNA lesions. The local relative dose will then decrease with increasing penetration depth in tissues. (**B**) For proton beams, the entrance dose is relatively low, followed by a sharp peak (the Bragg peak) in deeper tissues. Correspondingly, induced DNA damage will be most significant in these deeper tissues.

Treatment of patients with EBRT is done using a source outside the body and traditionally multiple doses of IR (fractions) are administered over the course of the treatment [42]. This reduces toxicity and allows critical cellular factors, such as cancer cell cycle phase, to redistribute between fractions [26]. Photon beams are the most widely used type of EBRT and have shown good efficacy for many cancer types. At the beginning of their track, when entering the body, they exhibit a high energy deposition, which reduces along the path and thus local radiation dose decreases with penetration depth (Figure 2A). For tumors located in deeper tissues, this energy deposition pattern might thus lead to a relatively high radiation dose at the entrance site of the body. This toxicity in healthy tissues is a well-known major drawback of EBRT [8]. However, by irradiating from different angles, a sufficient dose to the tumor can be reached while keeping radiation exposure to the healthy tissues minimal. Unfortunately, a wide variety of both early toxic effects, such as cell death in other highly proliferating tissues than the tumor, and late toxic effects, such as tissue fibrosis, have been reported. To counteract toxicity and improve therapy efficacy, the field of radiotherapy, especially photon therapy, has seen a large amount of developments with regard to radiation sources, treatment planning and delivery [26]. Notably, these include alterations of the ratio of dose per exposure and exposure frequency, known as hyper- or hypofractionation regimens.

In addition to photon beams, particle-based beams are increasingly incorporated in treatment schemes [28]. These beams, as opposed to γ-radiation, display an increasing energy deposition along their track, leading to a higher biological effect in deeper tissue regions [43] (Figure 2B). A sharp peak of the energy deposition occurs at the end of the particle track, termed the Bragg peak. Particle beam-based treatment, such as proton beam therapy (PBT), therefore can be most effective in deeper tumors, while sparing surrounding healthy tissue. It is suggested that PBT induces a high amount of complex DNA damage at the Bragg peak, although the exact nature of this damage, the mechanism by which this damage is induced and crucial repair pathways are not yet known [44]. When translating photon to proton therapy planning schedules in the clinic, an RBE of 1.1 is applied to adjust proton therapy dosage [31]. However, the RBE can significantly differ along the proton beam path, with important implications for therapy design [45]. This implies that more research into the exact effects of proton therapy is still crucial for further adaptation in the clinic.

#### 3.1.2. Brachytherapy

Brachytherapy is a form of internal radiotherapy where a radiation source (for example, an encapsulated radionuclide) is placed inside the body, close to the tumor [29]. Therefore, this form of therapy is mainly employed for patients with organ-confined cancer [46]. Brachytherapy has been used to treat several types of cancer, including those of the breast, prostate and cervix [29]. The two main methods are interstitial and intracavitary therapy [47,48]. During interstitial therapy, a radiation source is placed directly within the target tissue which, for example, is often used for breast cancer treatment. In contrast, intracavitary therapy includes placement of the source in close proximity to the target tissue, such as a nearby cavity. An important determinant in brachytherapy is the dose rate: during high dose rate (HDR) therapy, a radiation source, for example contained in a catheter, is placed inside the body temporarily and delivers a high radiation dose in a short timeframe. Low dose rate therapy (LDR) involves the permanent implantation of a source, often in the form of a seed. Currently, significant research efforts are aimed towards best practices in dose rate in various cancers [46]. For low-risk prostate cancer, for example, LDR brachytherapy has been utilized for years, but increasing data point towards HDR therapy as a superior therapy choice due to its lower acute and chronic toxicity [49].

There are important differences between anticancer effects of EBRT and brachytherapy, mainly based on the difference in distance between the radiation source and the tumor [29]. The center of a solid tumor seems to be targeted much more effectively by brachytherapy. Furthermore, the close proximity to the tumor of the radiation source leads to reduced toxicity in healthy tissues [50]. It is likely that a different tumor response will be induced by brachytherapy compared to EBRT, in terms of induced DNA damage and activated DDR components [51,52]. This difference will be highly dependent on total applied dose and dose rate.

A relatively novel form of brachytherapy that is used for both primary and metastasized liver cancer is radioembolization [53]. This therapy involves the intra-arterial administration of microspheres containing a radionuclide, mostly yttrium-90, which will accumulate in intratumoral capillaries due to their size distribution. The underlying rationale is that a relatively high radiation dose can be targeted to the tumor selectively [54], as the main blood supply of the tumor occurs via the liver artery. In contrast, blood supply for hepatocytes mainly occurs via the portal vein, limiting radiation exposure to healthy liver tissue.

#### 3.1.3. Molecular Radionuclide Therapy (MRT)

The principle of MRT relies on the systemic administration of a radioactive compound. The radionuclide can either physiologically be translocated to the tumor or is coupled to a tumor-targeting carrier small molecule, peptide or antibody, delivering a high radiation dose to the tumor specifically [55]. This strategy enables effective targeting of (metastasized) tumors while sparing healthy tissues. Nevertheless, hematological, renal and liver toxicities have been reported in MRT [56].

**Figure 3:**
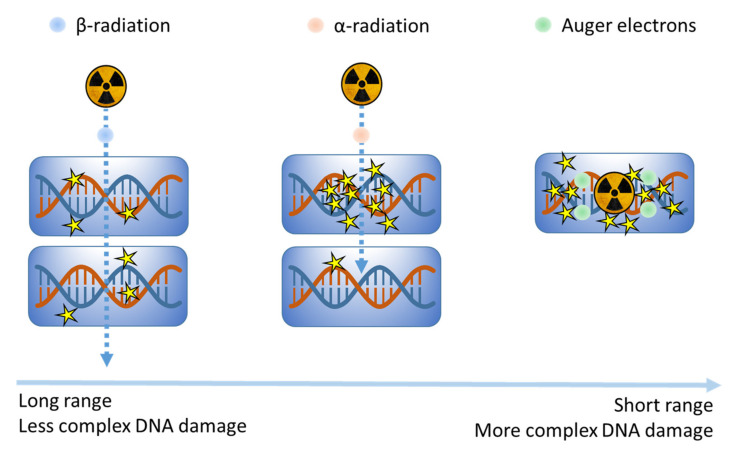
Visualization of DNA damage induction patterns of beta-emitters, alpha-emitters and Auger electrons, ordered (from left to right) by decreasing penetration range and increasing complexity of induced DNA damage. While beta-emitters exhibit a relatively long penetration range, they mostly induce isolated lesions. Alpha-emitters have a shorter range, but can locally induce more complex forms of DNA damage that are less readily repaired. Auger electrons display the shortest range of the three, but due to the fact that multiple electrons are released from the radionuclide they can still induce a high biological effect locally.

Various radionuclides are currently being clinically evaluated for use in MRT. These radionuclides emit different types of particles with varying energies, resulting in different LETs and penetration ranges and holding important implications for their biological effects [57,58]. These forms of radiation include α-particles, with a high LET and short range, inducing complex DSBs in close proximity to the source [59,60]. In contrast, β-particles have a lower LET and longer range, inducing less complex damage but enabling the targeting of larger tumors [58,61]. Finally, Auger electrons have a very short range due to their relatively low energy [57,61]. However, as multiple Auger electrons are released during radionuclide decay, they can exert a relatively high biological effect, inducing damage with high complexity. Due to their short range, Auger-emitters should be present in a cell nucleus to directly induce DNA damage [62]. A visualization of the biological effects of α-emitters, β-emitters and Auger electrons is depicted in Figure 3.

A classic example of MRT is the use of the β-emitter iodine-131, which is selectively taken up in the thyroid and thus can be effectively targeted to thyroid cancer [63]. Furthermore, β-emitter lutetium-177 coupled to the somatostatin analog octreotate is currently FDA and EMA-approved for the treatment of metastasized gastroenteropancreatic neuroendocrine tumors by targeting the overexpressed somatostatin receptor 2 [64]. In addition, of increasing importance is the coupling of radionuclides to prostate specific membrane antigen targeting molecules to treat prostate cancer, for which various research lines are currently ongoing towards a better toxicity profile and patient response [65].

Research on differences in DNA damage induction and relevant DDR pathways between MRT and EBRT is currently ongoing, with a major factor being the dose rate: while EBRT is applied as an acute high dose, local IR delivery by radionuclides can last for several days to weeks, depending on radionuclide half-life [66]. These features give important implications for our understanding of MRT radiobiology and future research directions. Apart from investigating different radionuclides, the current search for improvements includes testing other peptides or antibodies, identifying novel tumor target molecules and using combination treatments to enhance therapy effect [67].

### 3.2. Cytotoxic Chemotherapy

**Figure 4:**
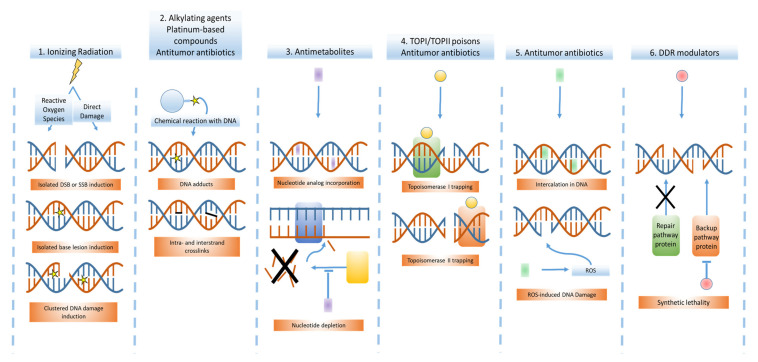
Visualization of the DNA-targeted mechanisms of action of radiotherapy, cytotoxic chemotherapy and DDR modulators. 1. Ionizing radiation (IR) can induce DNA damage both directly and indirectly (through ROS formation), leading to formation of SSBs, DSBs and different base lesions. Depending on the radiation type used, clustered (complex) DNA damage might be induced when multiple DNA lesions are formed in close vicinity. 2. Alkylating and platinum-based compounds harbor a reactive site and directly react with the DNA molecule., forming DNA adducts and intra- or interstrand crosslinks. 3. Antimetabolites mimic molecules essential in DNA replication and repair and, depending on the specific compound, can be incorporated in the DNA leading to DNA damage. Alternatively. antimetabolites can inhibit nucleotide producing pathways. 4. TOPI- and TOPII-poisons, the most clinically relevant topoisomerase inhibitors, trap topoisomerases on the DNA, preventing re-ligation of topoisomerase-induced breaks. For TOP1 poisons, DSBs are formed when DNA polymerase stalls on this trapped complex. However, for TOPII-poisons, trapping leads to the persistence of topoisomerase-induced DSBs. 5. Antitumor antibiotics can have different DNA-targeted mechanisms of action, such as compound intercalation in the DNA and induction of ROS formation. Other antitumor antibiotic mechanisms of action overlap with other cytotoxic chemotherapeutic classes, such as DNA alkylation and topoisomerase poisoning. 6. DDR modulators target specific DDR proteins and exert their cancer-inhibiting effect by synthetic lethality: as cancer cells that have loss-of-function mutations in specific DDR pathways become more reliant on backup repair pathways, inhibition of the latter by DDR modulators can specifically target cancer cells versus healthy cells.

Since the discovery of the anticancer properties of nitrogen mustards against lymphomas in the 1940s, many years of development in chemical biology have led to the widespread adaptation of cytotoxic chemotherapeutics in oncology [6,68]. The central principle of chemotherapy relies on the preferential targeting of highly proliferating cells by chemicals directly inducing DNA damage or interfering with DNA-related processes such as replication [69]. Furthermore, some chemotherapeutics, such as mitotic inhibitors, exert a mechanism of action that is not directly DNA-targeted. The five main classes of DNA-targeting cytotoxic chemotherapy, grouped by their mechanism of action, are alkylating agents, platinum-based compounds, antimetabolites, topoisomerase inhibitors and antitumor antibiotics [69]. Their respective mechanism of action is visualized in Figure 4.

Cytotoxic chemotherapies preferentially target tumor cells based on their rapid proliferation, as DNA damage induction and inability to repair this damage will be the most significant in these cells. However, a well-known major drawback of commonly used chemotherapeutics is their unfavorable toxicity profile, most profoundly in rapidly proliferating healthy tissues, with a wide variety of both early and late side-effects reported upon treatment. These range from acute toxicities such as nausea to more detrimental effects such as cardiotoxicity, bone marrow injury and the development of secondary malignancies [70,71,72]. Furthermore, and similar to radiotherapy, intrinsic or acquired resistance to chemotherapeutics remains a clinical challenge [9]. Resistance can occur on a macroscopic level, mainly by unfavorable absorption, distribution, metabolism and excretion drug properties. Furthermore, the cause of resistance can be on a molecular level, for example by altered transmembrane drug transport (e.g., increased efflux), inactivation of cell death pathways and elevated pro-survival signaling by oncogene activation or tumor suppressor gene inactivation [9]. Tumor-specific alterations of DDR signaling also significantly contribute to drug resistance, especially when they occur in crucial repair pathways for the DNA damage type induced by a given chemotherapeutic compound. For example, levels of RAD51, a major HR mediator, positively correlated with resistance to the chemotherapeutic etoposide in small cell lung cancer [73].

#### 3.2.1. Alkylating Agents and Platinum-Based Compounds

Alkylating agents react directly with the oxygen and nitrogen atoms of DNA bases to form a variety of adducts [74]. While monofunctional alkylating agents, such as temozolomide and dacarbazine, have a single reaction site forming an adduct on one DNA strand, bifunctional agents, such as aziridines and epoxides, can react with two strands simultaneously resulting in ICLs [75]. An important role in alkylating agent DNA damage repair is attributed to the BER and NER pathways, as well as to specific proteins that can directly remove the DNA adducts, mainly O-6-methylguanine-DNA methyltransferases (MGMT). In addition, various other pathways play a role in the processing of secondary lesions: MMR is activated upon DNA mispairing with an alkylated base and intermediate structures that arise during the repair of drug-induced lesions, such as DSBs, are repaired by HR and NHEJ [75,76].

Platinum-based compounds, such as cisplatin, carboplatin and oxaliplatin, exert a similar mechanism of action as alkylating compounds. After entering the cell, the compounds undergo hydrolysis, yielding a platinum-containing moiety with two DNA-reactive sites [77,78]. This process enables the molecule to form intra- and interstrand crosslinks. A multitude of repair pathways play a role in the removal of these DNA lesions [79]. Specifically, NER proteins are important in the removal of intrastrand crosslinks, and the HR, NER and FA pathways contribute to the removal of ICLs in a coordinated matter [80].

#### 3.2.2. Antimetabolites

In contrast to direct DNA reacting agents, antimetabolites function by mimicking molecules essential in DNA replication and repair [81]. These could either be compounds that inhibit deoxynucleoside triphosphate (dNTP)-producing pathways, depleting DNA polymerases of required nucleotides, or compounds that are incorporated in the DNA itself. Well-known examples are nucleoside analogs such as 5-fluorouacil (5-FU), which can both inhibit the synthesis of the nucleoside thymidine and, after metabolism of the drug into a nucleotide analog, can be incorporated in the DNA [82]. Depletion of necessary dNTPs leads to ineffective DNA damage repair and replication by termination of newly synthesized DNA segments [81]. Furthermore, incorporation of nucleotide analogs in the DNA can lead to chain termination. However, depending on the compound, chain elongation by DNA polymerases might be possible with the nucleotide analog erroneously incorporated. All of these mechanisms can result in activation of the DDR. The stalling of replication forks upon chain termination may lead to induction of SSBs, DSBs and other DNA lesions. Upon replication fork stalling, the activation of HR seems to play a crucial role in the cellular response to antimetabolites [83]. Alternatively, when nucleotide analogs are erroneously incorporated in the DNA, BER and MMR pathways have been described to be important repair pathways [84]. It was suggested that HR is also involved in the repair of intermediate structures during antimetabolite-induced BER and MMR repair [85].

#### 3.2.3. Topoisomerase Inhibitors

Topoisomerase inhibitors target topoisomerases, enzymes that counteract the over- or underwinding of DNA, for example during DNA replication [86]. The two main classes of these drugs are topoisomerase poisons, which stabilize the topoisomerase-DNA complex, and topoisomerase catalytic inhibitors, which inhibit enzymatic activity of topoisomerases by other mechanisms, such as preventing DNA binding [87]. Currently most clinically relevant topoisomerase inhibitors are poisons, which have been found to exert the most potent anticancer activity. Topoisomerase poisons act by binding protein-DNA complexes and trapping these complexes onto the DNA [88]. Type I topoisomerases (TOPI) poisons, such as topotecan and irinotecan, create SSBs to exert their function, while type II topoisomerase (TOPII), such as etoposide, create DSBs for this purpose. For TOPI-poisons, DSBs are formed when the DNA polymerase stalls upon the topoisomerase-DNA complex during replication [89]. While TOPII-induced DSBs are normally transient, binding of the topoisomerase-DNA complex by TOPII-poisons blocks re-ligation of these breaks, leading to damage persistence [90]. In both cases, the formation of DSBs will lead to induction of cell death. Because TOPI-poisons induce SSBs that can be converted into DSBs as a result from fork stalling during replication, both the BER and HR machinery are important in the cellular response to these compounds [91,92]. However, as TOPII-poisons induce DSBs throughout the cell cycle, HR and NHEJ can both be activated as a repair pathway [93,94].

#### 3.2.4. Antitumor Antibiotics

Antitumor antibiotics are a class of chemotherapeutics with various DNA-centered mechanisms of actions, partially overlapping with the chemotherapeutic classes described above. The main difference with the other chemotherapeutic classes is that antitumor antibiotics are derived from microbes such as *Streptomyces*. Important classes of antibiotics used in cancer care include anthracyclines, mitomycin C and bleomycin. Anthracyclines, with doxorubicin as a well-known example, both intercalate in the DNA and function as a TOPII-poison [95,96]. Additionally, these compounds generate high levels of ROS. However, the complete mechanism of action of doxorubicin is currently still unknown and a variety of other anticancer mechanisms have been proposed [97]. In contrast, mitomycin C functions as an alkylating compound by forming covalent linkages with the DNA [98]. Finally, bleomycins mimic the effects of IR by generation of ROS and subsequent DNA damage induction [99]. For this reason, these compounds are classified as a ‘radiomimetic’.

#### 3.2.5. Improvement of Chemotherapy

Compared to other rapidly evolving fields such as targeted therapies and immunotherapy, truly novel developments for cytotoxic chemotherapies are lagging behind. Few new cytotoxic chemotherapeutic drugs have reached the clinic in the past few years, with trabectedin, an alkylating compound that can interact with transcription, DNA repair and the tumor micro-environment, as one of the examples [100]. Rather, developments of chemotherapeutics have focused on the design of structural analogues of already approved compounds [69]. A good example is the design of cisplatin analogues, such as carboplatin, to improve drug properties, especially with regard to toxicity profiles [78]. Additionally, developments for cytotoxic chemotherapy include improved drug targeting and delivery methods, such as the use of liposomes and other nanocarriers and the use of pro-drugs that are activated by tumor-specific factors [101,102]. Furthermore, de-escalation of chemotherapeutic treatment, lowering treatment intensity to a point where the same beneficial clinical effect can be reached as when using the original dose, has gained a lot of attention for toxicity reduction [103]. Finally, a very important development, as for the other therapies described in this review, is the use of chemotherapeutics in combination regimens. This will be further explained below.

### 3.3. Targeted Therapies: Modulators of the DDR

While radiotherapy and cytotoxic chemotherapy target tumors through their relatively fast proliferation rate, the field of oncology has been shifting towards a form of medicine where tumor-specific factors can be targeted, leading to more favorable therapeutic windows [104]. These factors could be proteins that play a crucial role in the survival of tumor cells, while they are less important in healthy cells. Given the important role of the DDR in the cellular response to DNA damage and the fact that cancer cells often are genomically unstable and have inactivation of one or more DDR pathways, the development of drugs targeting DDR proteins has received considerable attention in the past few years [1]. These DDR modulators have been under investigation both as monotherapies and in combination with other DNA damaging therapies.

In many cases, especially in a monotherapy setting, the rationale behind the development of these DDR modulators is based on the concept of ‘synthetic lethality’: in cancer cells having loss-of-function mutations in DDR genes, consequently impairing a certain DDR pathway, the pharmacological inhibition of back-up pathways can lead to tumor-specific cell killing or proliferation inhibition [105]. Healthy cells that do not have this loss-of-function mutation are not dependent on these back-up pathways and will be able to repair the corresponding type of DNA damage via the original pathway. The textbook example of this concept is the inhibition of poly(ADP-ribose) polymerase (PARP) for breast cancer gene 1/2 (BRCA1/2)-deficient tumors [106]. Its underlying principle is that *BRCA1*- or *BRCA2*-deficiency leads to impaired HR in cancer cells, weakening the DSB repair capacity in these cells specifically. PARP inhibitors block the function of PARP1, an enzyme crucial in SSB repair [107]. Unrepaired SSBs will be converted to one-ended DSBs during replication, which require HR for repair. However, this is not possible due to the HR-deficiency of the cancer cells. In addition, PARP inhibitors can physically trap PARP onto the DNA, forming an obstacle for the replication fork and leading to fork stalling [108]. Collapse of stalled replication forks can consequently lead to additional DSB formation. The accumulation of DSBs during S-phase by PARP inhibitor treatment, in combination with impaired HR, results in a potent anticancer activity while healthy cells are not affected. Promising clinical results demonstrating this concept ultimately led to the approval of four different PARP inhibitors (olaparib, niraparib, rucaparib and talazoparib) for treatment of breast, ovarian and pancreatic cancer [109].

In addition to PARP inhibitors, several other DDR modulators are being evaluated. One of the strategies that has made the most clinical progress is inhibition of ataxia telangiectasia and Rad3-related (ATR) protein kinase, with currently several compounds in phase II clinical trials [110]. As a monotherapy, ATR inhibitors are effective in cancer cells with mutations in various proteins involved in DSB repair, including ATM [111]. ATR is essential in the response to replication stress and prevents the collapse of stalled replication forks, which would lead to DSB formation [112]. ATM mediates the initiation of DSB repair and is a central player in the activation of cell cycle checkpoints. Therefore, the proposed mechanism behind the synthetic lethal relationship between ATR and ATM is that ATR inhibition increases the number of DSBs and tumor-specific loss-of-function mutations in ATM impair the repair of these DSBs [113]. Similarly, synthetic lethal relationships of ATR and several proteins involved in HR have been found [114]. In addition, a variety of other DDR modulators are in clinical development and a vast amount of preclinical research currently focuses on the identification of novel potential drug targets, aided by the use of high throughput synthetic lethality screens [115].

Crucial to the use of DDR modulators, both in a monotherapy and combinatorial context, is the identification of patient-specific biomarkers. Detection tests should be able to accurately identify cancer-specific mutations that can be targeted through a synthetic lethal approach. Constant technical revolutions in patient stratification techniques such as next generation sequencing are resulting in different test types that can now guide treatment decisions [116]. For example, for PARP inhibitors, techniques identifying HR-deficient patients range from sequencing to functional ex vivo assays of DSB repair on tumor tissue [117]. Illustrating the need for patient stratification, BRACAnalysis CDx, a sequencing-based test for *BRCA1/2* mutations, has been FDA-approved as a companion diagnostic for PARP inhibitor treatment [118].

Despite the effectiveness of DDR modulators against a range of cancers, multiple resistance mechanisms have been reported. For PARP inhibitors, for example, these include increased drug efflux and reactivating mutations in *BRCA1/2* genes [119]. An important strategy to counteract resistance could be the combination of DDR modulators with other DNA damage-inducing therapies, which will be further described below.

## 4. Combination Approaches Involving DNA Damage-Inducing Therapies

The combination of different anticancer modalities is seen as an important future direction for cancer treatment and this is also a significant area of interest for DNA damage-inducing therapies [120,121]. The use of combination treatments has several potential advantages over monotherapy application, mainly increased anticancer efficacy and reduced toxicity. The latter can occur when two or more drugs can be used at a lower concentration than when they would be administered as a monotherapy. Moreover, if the used agents target different cellular pathways, development of acquired resistance can be delayed or prevented.

Clinically relevant combination therapies can show an additive effect, where the effect of the combination is merely the sum of the effects of the independent DNA damaging agents [122]. This occurs when the components of a combination regimen induce two mechanistically separate forms of DNA damage. However, ideally, the combination of two given agents is synergistic, where the effect of multiple drugs combined is greater than the sum of their individual effects. Synergistic effects can result from interactions between two or more drugs on multiple levels, including that of the DDR. Importantly, to exploit the advantages of combination therapies, a rational method of combination design is necessary, based on extensive knowledge of therapy mechanism of action including activated DDR pathways.

In this section we will summarize the main combinatorial approaches for DNA damage-inducing therapies, as well as combinations of those therapies with immunotherapy.

### 4.1. Radiotherapy and Chemotherapy Combinations

One of the oldest combination strategies in oncology is that of combining multiple cytotoxic chemotherapies, which was already put into practice in the 1950s [123]. Nowadays, chemotherapeutic combinations are still very effective treatments for various malignancies, such as childhood acute lymphoblastic leukemia [124]. Over the years, many chemotherapeutic combinations have been developed showing a potent antitumor effect through additive effects. However, synergistic combinations of chemotherapeutics can be achieved when the DNA damaging mechanisms of individual compounds can amplify each other. A notable example of a chemotherapeutic class that is used in several successful chemotherapy combinations are antimetabolites [125,126]. By interfering with nucleotide synthesis and their incorporation in newly synthesized DNA, these compounds can inhibit the repair of DNA damage induced by other chemotherapeutics [125]. An example is the introduction of a combination regimen containing oxaliplatin, irinotecan and the antimetabolite 5-FU (FOLFIRINOX) for the treatment of metastatic pancreatic cancer. Although this treatment was unfortunately associated with increased toxicity, its development marks one of the most significant steps forward in treating this malignancy up to this date [127].

Several years after the first chemotherapy combinations, the combination of radiotherapy and chemotherapy was introduced in the clinic [128]. In addition to the fact that these regimens increased antitumor efficacy as compared to radiotherapy alone for various cancers, they provide the opportunity for spatial cooperation; while radiotherapy can be used for treatment of local tumors, systemic chemotherapy can be used for (distant) metastases [129]. Similar to chemotherapy combinations, DDR-mediated cellular responses to radiotherapy- and chemotherapy-induced DNA damage can interact in various ways, leading to potent combination therapies. One example is the combination of cisplatin with radiotherapy, which is now successfully being used for treatment of, amongst others, cervical cancer [130]. Cisplatin treatment leads to the formation of DNA adducts that can block repair of nearby IR-induced DNA breaks, resulting in damage persistence and cell death [131]. Furthermore, the effectiveness of antimetabolites in combination therapies has also been shown in combination with radiotherapy. In this case, antimetabolites can inhibit repair of IR-induced DNA damage. For example, 5-FU is used in combination with radiotherapy for the treatment of gastrointestinal tumors [132].

Finally, the combination of different types of radiotherapy is commonly applied, with the combination of EBRT and brachytherapy as a notable example. In the treatment of cervical cancer, this regimen was shown to result in increased cancer-specific survival and overall survival as compared to EBRT alone [133]. The use of brachytherapy in addition to EBRT has the advantage of delivering a high local dose to the tumor specifically.

A challenge in developing radiotherapy or chemotherapy combinations is selecting the right therapy timing and sequence, since this has implications for both efficacy and toxicity. It is important to compare between concomitant versus sequential administration of multiple agents. For example, combinations of radiotherapy and chemotherapy led to better patient outcome in locally advanced non-small cell lung cancer when administered concomitantly, although this was associated with increased toxicity as compared to sequential administration [134]. The most effective therapy sequence strategy is highly dependent on the used agents, dose and specific disease indication and thus requires optimization for specific cases. A second challenge is the identification of biomarkers to select patients for specific combinations, which could be cancer-specific DDR defects. As an example, combining temozolomide with radiotherapy for glioblastoma treatment led to improved outcome as compared to radiotherapy alone, but only in tumors with a silenced gene for MGMT [135]. The underlying mechanism is that MGMT is involved in the removal of DNA alkylations by temozolomide. A functional MGMT protein might thus counteract the radiosensitization by temozolomide by repairing temozolomide-induced DNA damage.

### 4.2. DDR Modulator Combinations

In addition to DDR modulator monotherapy treatments, of which efficacy mainly depends on synthetic lethal relationships with cancer-specific DDR mutations, combinations with other DNA damage-inducing therapies are actively being investigated [1]. Currently most data on DDR modulator combination regimens is available for PARP inhibitors, but other compounds such as ATR inhibitors are gaining increasing attention [136]. An important condition for these combination therapies is that the DDR modulator impairs a pathway involved in repair of the induced DNA damage by radiotherapy or chemotherapy, thereby leading to damage persistence and increase of cell death. Demonstrating this concept, inhibitors of DNA-PK are investigated as a sensitizing agent for radiotherapy and chemotherapy [137]. DNA-PK is an important player in NHEJ. Unfortunately, clinical trials of combinations of DNA damaging therapies with DDR modulators resulted in unfavorable toxicity profiles [1]. For example, combining PARP inhibitors with chemotherapy resulted in overlapping bone marrow toxicity [138,139]. This underlines the necessity for more detailed insight in dosing and timing schemes to allow further development.

Cancers that harbor DDR mutations might also benefit from targeting multiple DDR components: for example, inhibition of ATR after PARP inhibitor therapy can overcome PARP inhibitor resistance in tumors with a *BRCA*-mutant genetic background [140]. In addition, using multiple DDR modulators can induce an artificial form of synthetic lethality in cancers without DDR mutations that can be exploited by DDR modulator monotherapies: simultaneous application of multiple DDR modulators can impair both a repair pathway and a back-up pathway at the same time [141]. In the absence of specific DDR defects in the tumor, it is the intrinsically higher amount of DNA damage due to genomic instability in cancer cells as compared to healthy cells that will result in a potentially acceptable therapeutic window. The independence of these combinations on specific DDR mutations could make them applicable to a larger patient population than other DDR modulator-based therapies. However, similar to combinations of DDR modulators with chemotherapy, a potential drawback of combining multiple DDR modulators are the overlapping toxicity profiles, especially with regard to bone marrow toxicity [142]. Thus, determining optimal dosing, timing and sequencing of these combination therapies is crucial.

### 4.3. Immunotherapy Combinations

Few therapies in oncology have seen such tremendous development during the past two decades as immunotherapy [143]. Nowadays immunotherapy is positioned as one of the major pillars of cancer treatment, among surgery, radiotherapy and chemotherapy. Generally, this group of therapies works by empowering the body’s own immune system to eradicate cancer cells. However, tumors employ multiple cellular mechanisms to evade recognition and cell killing by immune system components, of which one is the upregulation of immune checkpoint molecules that bind and inhibit adaptive immune cells [144]. The application of antibodies against these molecules, called immune checkpoint inhibitors, can significantly counteract this form of immune evasion [145].

DNA-damaging therapies have the ability to potentiate the immune response to kill cancer cells [146,147]. Currently multiple clinical trials are running that investigate combinations of immune checkpoint inhibitors with radiotherapy, chemotherapy and DDR modulators. Importantly, treatment with DNA damaging agents can lead to immunogenic cell death, resulting in release of tumor-specific antigens and, among other factors, danger-associated molecular pathogens (DAMPs) in the environment [148]. Both these antigens and DAMPs can stimulate the adaptive immune response. However, the relationship between tumor DNA damage, DNA damage repair and the immune system is complex. Currently a wide variety of other potential mechanisms by which therapy-induced DNA damage and repair influence the anticancer immune response are under investigation (reviewed in [149]). An example is the activation of the stimulator of interferon genes pathway in the tumor cell by cytosolic DNA fragments after DNA damage induction. This will lead to transcription of pro-inflammatory interferon genes, stimulating the immune response [150].

In addition to investigating the mechanisms of immunogenicity of DNA damage-inducing therapies, current research focuses on selection of suitable agents to combine with immunotherapy and optimal dosing, sequence and timing [147]. Furthermore, identification of patient-specific biomarkers to predict therapy efficacy for these combination regimens is a major focus point. Given the interplay between DNA damage and repair and immunotherapy, cancer-specific DDR defects are currently considered as potential valuable biomarkers [149].

## 5. Conclusions

DNA damage-inducing therapies have been of great importance for cancer treatment for decades. The DDR is a crucial network in the cellular response to these therapies, guiding the decision between repair of induced DNA lesions or induction of detrimental cellular fates such as cell death. Additionally, the DDR plays a major role in both intrinsic and acquired therapy resistance. A large amount of research efforts has led to an improved understanding of DNA damage-inducing drug properties and the DDR pathways that mediate resulting cellular responses.

Currently a multitude of developments are predicting the important role for DNA damage-inducing treatments in oncology to continue. Next to ongoing developments for the ‘classical’ therapies, radiotherapy and cytotoxic chemotherapy, the introduction of targeted drugs in oncology has found its way into the field of DNA damage-inducing therapies through the development of DDR modulators. Furthermore, combination therapies are an important future direction in oncology, as they can increase anticancer efficacy of other treatments compared to monotherapy and lower the chance of development of resistance. It is important to note that in some cases the pronounced cytotoxic effects of radiotherapy and chemotherapy can be indispensable to achieve a potent and durable anticancer response in combination therapies. Moreover, radiotherapy and cytotoxic chemotherapy are still a very effective first line treatment for a large variety of malignancies. It is therefore unlikely that targeted therapies and other regimens such as immunotherapy will completely replace radiotherapy and cytotoxic chemotherapy in the near future, but rather it is the careful selection, based on a specific cancer genotype or phenotype, or combination of these modalities that is of great potential for cancer treatment.

Important future challenges for DNA damage-inducing therapies include the further elucidation of therapy mechanisms of action and crucial components of the DDR that mediate the cellular response to therapy. A more detailed understanding of these properties can guide selection of DNA damaging agents and treatment schemes for specific cancers. Notably, the improved understanding of cellular therapy responses aids in the rational development of novel combination regimens of DNA damage-inducing agents, potentially also with other therapy types such as immunotherapy. This knowledge is also crucial for identifying biomarkers to guide patient selection for specific agents. Although for DNA damaging agents these biomarkers are often DDR-based, other cellular factors such as tumor proliferation rate might also play a role here. The identification of biomarkers not only has the potential to personalize targeted therapies, but can also be used to refine radiotherapy and chemotherapy. Ultimately, adaptation of treatment schemes to specific patients could lead to further improvement of therapeutic windows.
